# A germline-targeted genetic screen for *xrn-2* suppressors identifies a novel gene *C34C12.2* in *Caenorhabditis elegans*

**DOI:** 10.1590/1678-4685-GMB-2022-0328

**Published:** 2023-05-15

**Authors:** Ilkin Aygün, Alicja Rzepczak, Takashi S. Miki

**Affiliations:** 1 Polish Academy of Sciences, Institute of Bioorganic Chemistry, Department of Developmental Biology, Poznań, Poland.

**Keywords:** XRN2, C34C12.2, glycerol regulation, mutagenesis screen, germline development

## Abstract

XRN2 is an evolutionarily conserved 5’-to-3’ exoribonuclease, which degrades or trims various types of RNA in the nucleus. Although XRN-2 is essential for embryogenesis, larval development and reproduction in *Caenorhabditis elegans*, relevant molecular pathways remain unidentified. Here we create a germline-specific *xrn-2* conditional mutant and perform a mutagenesis screen for suppressors of sterility. Loss-of-function alleles of *dpy-10*, *osr-1*, *ptr-6* and *C34C12.2* genes are identified. Depletion of DPY-10, OSR-1 or PTR-6 increases expression of *gpdh-1* that encodes a glycerol-3-phosphate dehydrogenase, thereby elevates glycerol accumulation to suppress sterility of the mutant. The C34C12.2 protein is predominantly localized in the nucleolus of germ cells and shows a similarity to *Saccharomyces cerevisiae* Net1, which is involved in rDNA silencing. Depletion of NRDE-2, a putative interacting partner of C34C12.2 and a component of the nuclear RNAi machinery, restores fertility to the *xrn-2* conditional mutant. These results may help to identify an essential role of XRN-2 in germline development.

## Introduction

XRN2 is an evolutionarily conserved 5’-to-3’ exoribonuclease. Predominantly localized in the nucleus, it degrades or trims various types of RNA for their maturation, level control or surveillance (Miki and Großhans, 2013; Nagarajan *et al*., 2013). Targets of XRN2 include the precursor, mature or aberrant forms of rRNA, tRNA, mRNA and microRNA (miRNA). Among multiple functions of XRN2, its role in rRNA maturation has been well-characterized in many species including yeast (Amberg *et al*., 1992; Petfalski *et al*., 1998; Fang *et al*., 2005), ciliates (Couvillion *et al*., 2012), kinetoplastids (Sakyiama *et al*., 2013), plants (Zakrzewska-Placzek *et al*., 2010) and mammals (Wang and Pestov, 2011). Precursor rRNA (pre-rRNA) is transcribed as a single molecule by RNA polymerase I in the nucleolus. During its processing into three rRNA species, XRN2 plays crucial roles in maturation of 5.8S and 25S/28S (in yeast/mammals, respectively) rRNAs by trimming the 5’ ends of their precursors. 

We have previously shown that *xrn-2* is ubiquitously expressed throughout the development of *Caenorhabditis elegans* (*C. elegans*) and that its activity is required for embryogenesis, larval development and reproduction (Miki *et al*., 2014a ). Although XRN-2 has been reported to degrade miRNA (Chatterjee and Grosshans, 2009; Miki *et al*., 2014a,b) and pre-mRNA (Miki *et al*., 2016) and to terminate transcription by RNA polymerase II on a subset of genes (Miki *et al*., 2017) in *C. elegans*, it remains unclear whether these functions are required for development. Ubiquitous expression and the multifunctional nature of XRN-2 make it difficult to relate one molecular pathway to one developmental process. Genetic screens for enhancers or suppressors of each developmental phenotype are expected to address the issue.

Here we create a germline specific *xrn-2* conditional mutant and perform a genetic screen for suppressors of sterility. Loss- or reduced-function alleles of *dpy-10*, *osr-1*, *ptr-6* and *C34C12.2* genes are recovered. Depletion of DPY-10, OSR-1 or PTR-6, but not of C34C12.2, increases accumulation of glycerol, leading to restoration of fertility to the mutant animals. C34C12.2 is predominantly localized in the nucleolus of germ cells and partially homologous to *Saccharomyces cerevisiae* Net1, which has a role in rDNA silencing. Depletion of NRDE-2, a putative interacting partner of C34C12.2 and an effector protein in the nuclear RNAi pathway, suppresses sterility of the *xrn-2* mutant, indicating that the two proteins might function together to counteract the role of XRN-2 in germline development.

## Material and Methods

### Worm strains

The Bristol N2 strain was used as wild type. The mutant strains used are listed in Table S1.

### Worm culture

Worms were cultured on Nematode Growth Medium (NGM) agar seeded with Escherichia coli OP50 according to the standard methods described previously (Brenner, 1974).

### Single copy transgene insertion

Mos1-mediated single-copy transgene insertion (MosSCI) was performed as described previously (Frøkjaer-Jensen *et al*., 2008). Insertion loci are shown in Table S1.

### Mutagenesis screen

About 4,000 *xrn-2ts* ^*germ*^ animals at the fourth larval stage were harvested, washed and incubated with 50 mM ethyl methanesulfonate (EMS) in 6 ml of M9 buffer for 4 hours at 20 °C. The worms were washed three times with M9 buffer and incubated at 20 °C. Once many eggs appeared on the plate, P0 animals were moved to fresh plates. This process was repeated for 2 days. Once many larvae of the F2 generation appeared, the animals were harvested, from which F1 animals were removed by brief centrifugation. The mutated F2 larvae were incubated at 25.5 °C and screened for fertility. Gravid animals were isolated and backcrossed 5 times with the parental *xrn-2ts* ^*germ*^ strain to remove unrelated mutations. Whole genome sequencing of the animals was performed as described previously (Miki *et al*., 2017). Mutations were mapped by single nucleotide variant analysis using the MiModD software (Baumeister Lab., University of Freiburg, Germany) according to the guideline.

### Microscopy

Stereoscopic images were obtained with an M205A stereo microscope (Leica, Solms, Germany) or an SMZ25 stereo microscope (Nikon, Tokyo, Japan). DIC and fluorescent images were obtained using an Axio Observer Z1 microscope (Carl Zeiss, Oberkochen, Germany).

### RNAi

RNAi clones were obtained from the Ahringer library (Kamath and Ahringer, 2003). RNAi was performed by the feeding method (Timmons and Fire, 1998) with bacteria carrying the insertless L4440 RNAi vector as a negative control.

### Quantitative reverse transcription PCR (RT-qPCR)

Worms were harvested, washed three times with M9 medium, resuspended in 700 µl of TRIzol Reagent (Thermo Fischer Scientific, Waltham, MA, USA) and frozen in liquid nitrogen. They were broken open by five repeats of freeze and thaw using liquid nitrogen using 42 °C heating block, before RNA was extracted and purified by the Direct-zol RNA MiniPrep Kit (Zymo Research, CA, USA) according to the supplier’s protocol. cDNA was generated from total RNA by the High-Capacity cDNA Reverse Transcription Kit with oligo(dT)18 Primer (Thermo Fischer Scientific) according to the supplier’s protocol. RT-qPCR was performed by QuantStudio 3 Real-Time PCR system (Thermo Fischer Scientific) using PowerUp SYBR Green Real-Time PCR Master Mix (Thermo Fischer Scientific) and specific primers for *gpdh-1* (sense: GCAATTGTTGGCGGTGGAAACTGG, antisense: CCTGGTTTCCTGGAATCTCTGCAC) and *act-1* (sense: AAATCACCGCTCTTGCCCCATCAA, antisense: GCACTTGCGGTGAACGATGGAT).

## Results

### Creation of a germline-specific *xrn-2* conditional mutant

**Figure 1 -:**
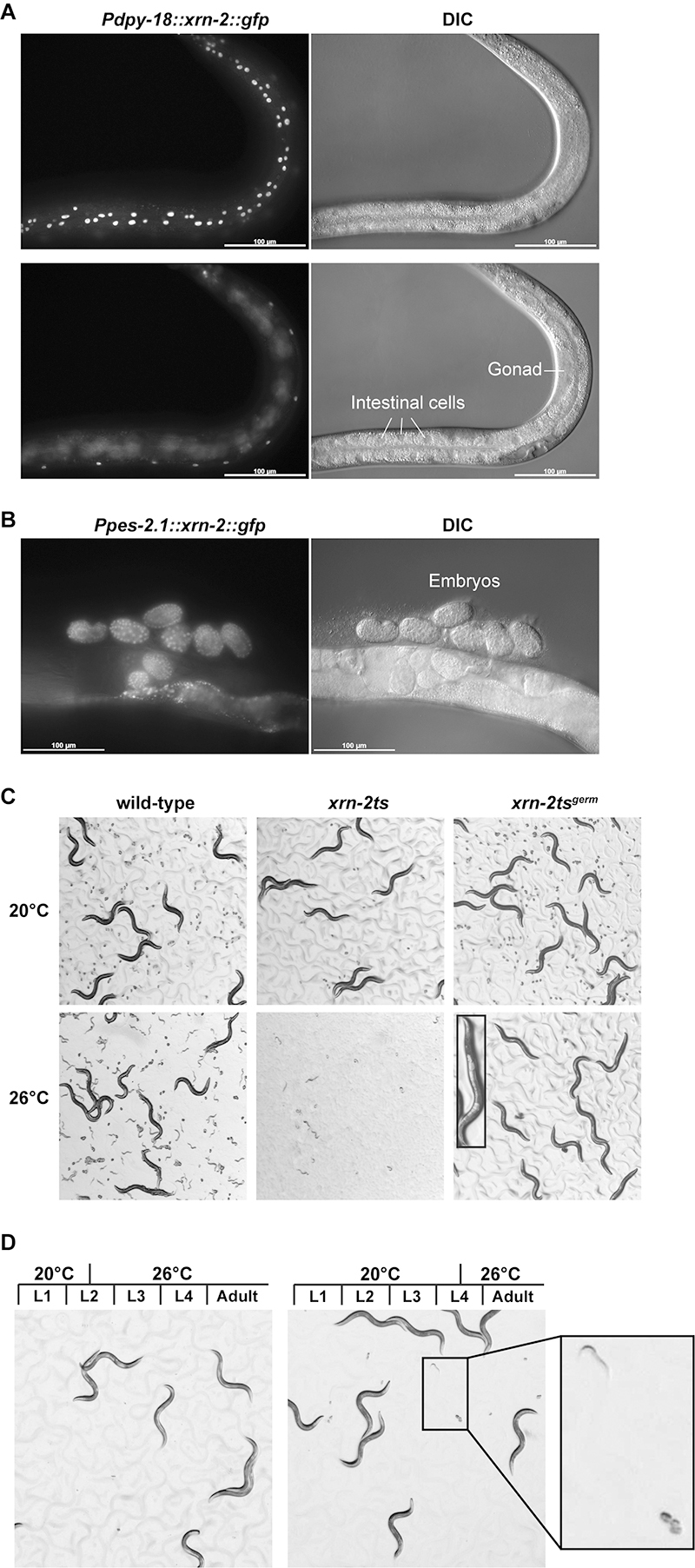
Creation of a germline-specific xrn-2 temperature-sensitive mutant. (A) Animals with a *dpy-18* promoter-driven *xrn-2::gfp* transgene (*Pdpy-18::xrn-2::gfp*) were incubated at 20 °C and observed. GFP signal was detected in hypodermal cells (top) but not in the intestine or the gonad (bottom). Corresponding DIC images are shown (right). (B) Animals with a *pes-2.1* promoter-driven *xrn-2::gfp* transgene (*Ppes-2.1::xrn-2::gfp*) were incubated at 20 °C and observed. GFP signal was detected in embryos. Corresponding DIC images are shown right. (C) Wild-type, *xrn-2ts* and *xrn-2ts* ^*germ*^ animals were incubated at 20 °C or 26 °C from L1 stage for 72 hours and observed by stereomicroscopy at the same magnification. An inset shows an adult animal without embryos at higher magnification. (D) *xrn-2ts* ^*germ*^ animals were incubated at 20 °C from L1 stage until the middle of L2 (left) or L4 (right) stage, then at 26 °C until the adult stage. The animals were observed by stereomicroscopy at the same magnification. An inset shows embryos and a hatched larva.

In order to gain more insight into roles of XRN-2 in *C. elegans* development, we decided to perform genetic screens for suppressors of developmental defects caused by inactivation of XRN-2. A conditional mutant is a powerful tool, which enables screening for genetic modifiers of an essential gene. We have previously reported temperature-sensitive alleles of *xrn-2* in *C. elegans* (Miki *et al*., 2014a , 2016, 2017). The mutant animals showed defects in many developmental processes including embryogenesis, larval development and fertility at restrictive temperatures. Genetic suppressor screens using these mutants had failed to identify any mutations other than those in *xrn-2* itself. Since *xrn-2* is expressed ubiquitously throughout development (Miki *et al*., 2014a), we reasoned that a single allele might not be able to suppress all developmental defects in different tissues or cells of *xrn-2* mutant animals. Therefore, to narrow down the target of screening, we focused on germline development. In order to create a germline specific *xrn-2* temperature-sensitive mutant (*xrn-2ts* ^*germ*^ ), we expressed a green fluorescent protein (GFP)-fused functional *xrn-2* transgene (*xrn-2::gfp*) in somatic cells of *xrn-2(xe31)*, a temperature-sensitive mutant of *xrn-2* (*xrn-2ts*) (Miki *et al*., 2017), by MosSCI (Frøkjaer-Jensen *et al*., 2008). For rescue of somatic cells, we expressed *xrn-2::gfp* using the promoter of *dpy-18*. XRN2-GFP signal was detected in hypodermal and other somatic cells, but not in the intestine or the gonad (Figure 1A). For rescue of the embryo, we expressed *xrn-2::gfp* using the promoter of *pes-2.1*, which is active specifically in the embryo (Figure 1B). When incubated at a permissive temperature (20 °C) from the first larval (L1) stage, the *xrn-2ts* ^*germ*^ animals developed to adult and reproduced. At a restrictive temperature (26 °C), on the other hand, *xrn-2ts* ^*germ*^ animals developed to adult but were sterile, while *xrn-2ts* animals ceased development as larvae (Figure 1C), as previously reported (Miki *et al*., 2017). Thus, the *xrn-2ts* ^*germ*^ strain can function as a tool to screen for genetic suppressors of sterility caused by XRN-2 inactivation in the germline. In order to identify developmental stages that require XRN-2 for fertility, we applied different timings of temperature shifts to *xrn-2ts* ^*germ*^ animals. The animals were fertile, when temperature was elevated from the middle of L4 stage, though not of L2 stage (Figure 1D). These results indicate that XRN-2 plays a crucial role in germline development before the mid-L4 stage.

### ***dpy-10*, *osr-1*, *ptr-6* and *C34C12.2* genes are identified as genetic suppressors of *xrn-2ts*** ^*germ*^

**Figure 2 -:**
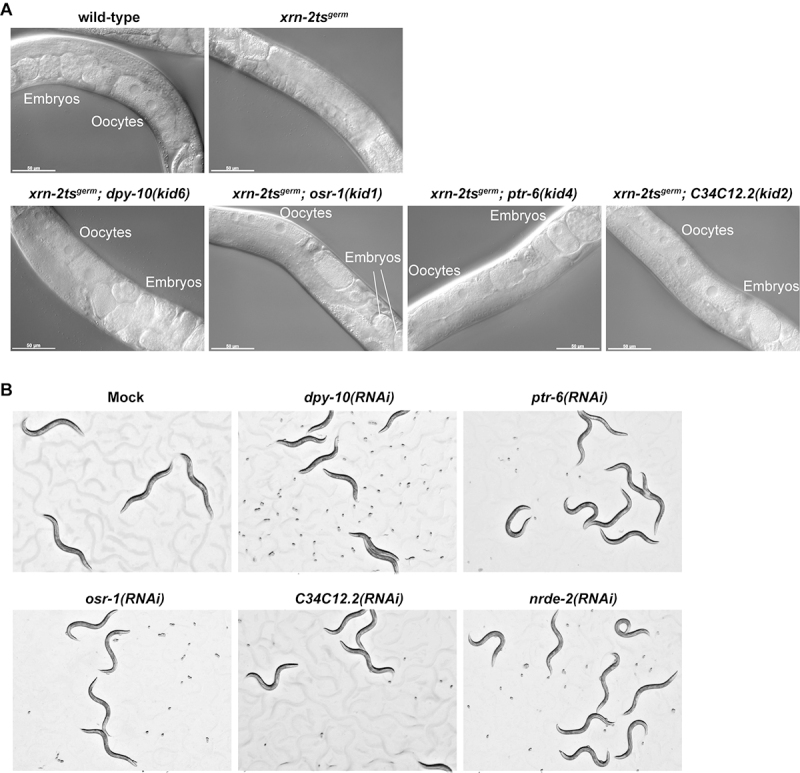
*dpy-10, osr-1, ptr-6* and *C34C12.2* genes were identified as genetic suppressors of *xrn-2tsgerm*. (A) Animals of indicated genotypes were incubated at 25.5 °C from the L1 stage for 72 hours and observed. Oocytes and embryos were found in all strains except *xrn-2ts* ^*germ*^ . (B) *xrn-2ts* ^*germ*^ animals were exposed to mock RNAi or RNAi for indicated genes from L1 to adult at 25.5 °C and observed by stereomicroscopy at the same magnification. Embryos were found in all conditions except mock RNAi.

We mutagenized *xrn-2ts* ^*germ*^ animals with EMS and isolated four strains that were able to reproduce at 25.5 °C (Figure 2A). Genomic DNA sequencing of these strains followed by mutation mapping identified recessive alleles of *dpy-10*, *osr-1*, *ptr-6* and *C34C12.2* genes (Table 1). *dpy-10* encodes a collagen protein in the cuticle (Levy *et al*., 1993). The *dpy-10(kid6)* allele has a missense mutation, and the mutant animals showed a dumpy phenotype. *osr-1* was identified as a gene whose loss conferred resistance to osmotic stress on animals (Solomon *et al*., 2004), and the *osr-1(kid1)* allele has a nonsense mutation. *ptr-6* encodes a member of patched family proteins, and the *ptr-6(kid4)* allele changes an amino acid in an evolutionarily conserved extracellular ligand binding site (Kuwabara and Labouesse, 2002; Zugasti *et al*., 2005; Daggubati *et al*., 2022). Although we recovered another allele of *ptr-6* with a missense mutation that substituted glycine at amino acid position 635 to glutamic acid, the strain carrying the allele was permanently lost due to an extreme difficulty in cryopreservation as previously reported (Choi *et al*., 2016). *C34C12.*2 encodes a protein of unknown function, and the *C34C12.2(kid2)* allele changed the guanine at the 5’ splice site of the fourth intron to alanine, abrogating splicing. Thus, these alleles were expected to reduce or abolish the gene functions. Consistently, RNAi-mediated knockdown of each of the genes restored fertility to *xrn-2ts* ^*germ*^ animals at a restrictive temperature (Figure 2B). These results suggest that the four genes counteract the function of *xrn-2* in germline development directly or indirectly.

**Table 1 -:** Alleles recovered from the screen.

Gene(allele)	Type of mutation	Nucleotide change	Codon change	Amino acid change
*dpy-10(kid6)*	Missense	G → A	GGA → AGA	G131R
*osr-1(kid1)*	Nonsense	C → T	CAA → TAA	Q239Stop
*ptr-6(kid4)*	Missense	G → A	GGA → GAA	G223E
*C34C12.2(kid2)*	Splice site	G → A^*^	n/a	n/a

^*^ 5’ splice site of intron 4

n/a: not applicable

### DPY-10, OSR-1 and PTR-6 control glycerol accumulation

**Figure 3 -:**
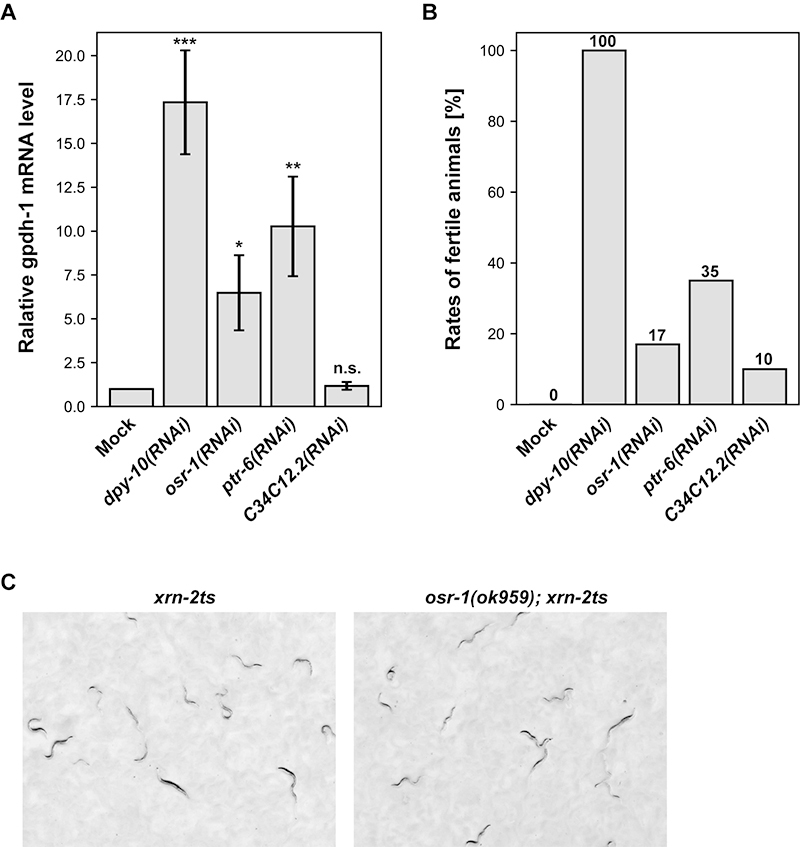
Knockdown of *dpy-10, osr-1, ptr-6* or *C34C12.2* gene increases *gpdh-1* expression. (A) Wild-type animals were exposed to mock RNAi or RNAi for indicated genes from L1 to adult at 25 °C. Levels of *gpdh-1* mRNA were quantified by RT-qPCR and normalized to *act-1* mRNA levels with values of mock-treated animals defined as 1 (n = 5, means ± SEM). p-values were calculated according to the two-sided paired t-test and marked: *p < 0.05, **p < 0.01, ***p < 0.001, n.s. not significant. Values are shown in Table S2. (B) *xrn-2ts* ^*germ*^ animals were exposed to mock RNAi or RNAi for indicated genes from L1 to adult at 26 °C, and rates of fertile animals were examined (n = 100 in each condition from two independent experiments). (C) Animals of indicated genotypes were incubated at 25 °C from L1 stage for 72 hours and observed by stereomicroscopy at the same magnification.

We became aware from literature searches that *dpy-10*, *osr-1* and *ptr-6* were among positive genes in a genome-wide RNAi screen for activation of the promoter of *gpdh-1*, a glycerol-3-phosphate dehydrogenase that mediates glycerol synthesis (Lamitina *et al*., 2006). Consistently, RNAi-mediated knockdown of *dpy-10*, *osr-1* or *ptr-6* increased *gpdh-1* mRNA levels (Figure 3A), which correlated roughly with the rates of animals that restored fertility (Figure 3B). Thus, DPY-10, OSR-1 and PTR-6 negatively regulate *gpdh-1* expression, and depletion of each of them de-represses *gpdh-1* to elevate glycerol levels, leading to restoration of fertility to *xrn-2ts* ^*germ*^ animals. Our attempt to restore fertility to *xrn-2ts* ^*germ*^ animals by providing glycerol externally from culture plates failed, possibly because the animals were reluctant to take exogenously provided glycerol (Figure S1).

The *xrn-2ts* and *xrn-2ts* ^*germ*^ strains have the *xrn-2(xe31)* allele that has a missense mutation to destabilize XRN-2 at elevated temperatures (Miki *et al*., 2017). Since glycerol is known to stabilize proteins (Vagenende *et al*., 2009), we suspected that the *dpy-10*, *osr-1* and *ptr-6* alleles stabilized mutant XRN-2 in the germ cells of *xrn-2ts* ^*germ*^ animals at the elevated temperature, leading to restoration of fertility. Indeed, *dpy-10* alleles have been reported to suppress phenotypes of several temperature-sensitive mutants (Maine and Kimble, 1989; Goh and Bogaert, 1991; Nishiwaki and Miwa, 1998; O’Rourke *et al*., 2007). If this is the case, elevated accumulation of glycerol, as it diffuses throughout the animal body, should be able to stabilize mutant XRN-2 not only in germ cells but in somatic cells of *xrn-2ts* animals to support their development. However, knockdown of *dpy-10*, *osr-1* or *ptr-6* failed to rescue the *xrn-2ts* animals from larval arrest (Figure S2). Since RNAi-mediated depletion of the target protein is usually partial, we crossed *osr-1(ok959)*, a loss-of-function allele of *osr-1* (Wheeler and Thomas, 2006), into *xrn-2ts* (*osr-1(ok959); xrn-2ts*), to examine the effect of constitutive deletion of the gene. When incubated from the L1 stage at 25 °C, all *osr-1(ok959); xrn-2ts* animals ceased development as larvae and showed no developmental advantage over *xrn-2ts* animals (Figure 3C). These results indicate that *gpdh-1* upregulation restored fertility to *xrn-2ts* ^*germ*^ animals by other means than stabilizing mutant XRN-2 at the elevated temperature.

### C34C12.2 is predominantly localized in the nucleolus of germ cells

**Figure 4 -:**
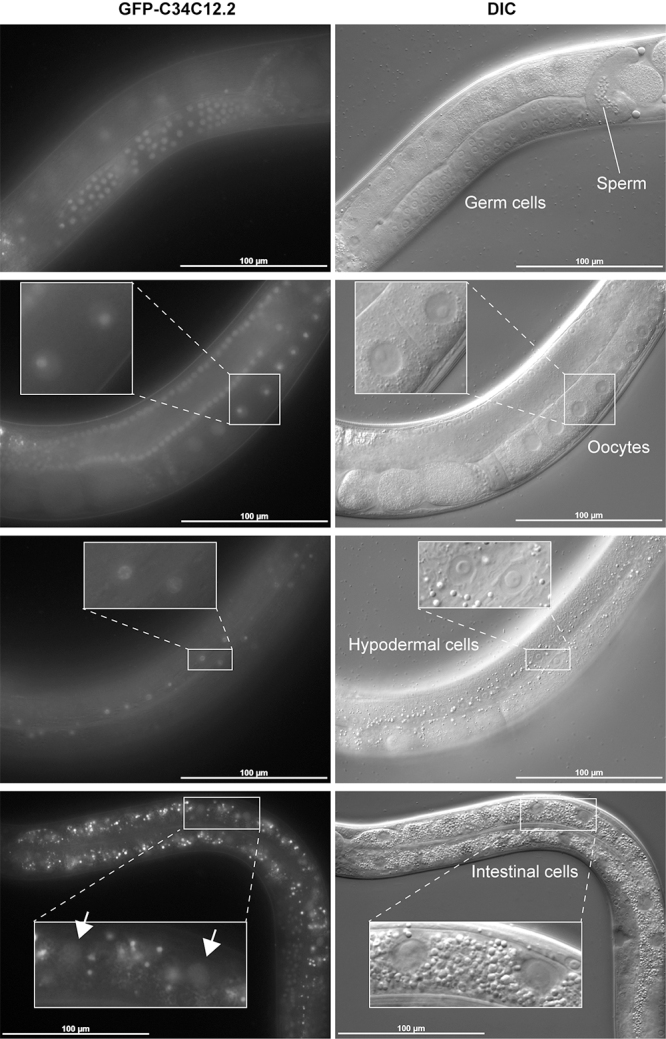
*C34C12.2* is predominantly localized in the nucleolus. Animals with a *gfp::C34C12.2* transgene were incubated at 20 °C and observed. Insets show cell nuclei at higher magnification. GFP signal was detected in the nucleolus of germ cells, oocytes, sperm, hypodermal cells and intestinal cells. Corresponding DIC images are shown right. Punctate signal in the intestine is the autofluorescence of gut granules (Coburn and Gems, 2013)

In contrast to three other genes identified in the screen, knockdown of *C34C12.2* did not affect *gpdh-1* expression (Figure 3B). Thus, the *C34C12.2* allele is likely to restore fertility to *xrn-2ts* ^*germ*^ animals through a mechanism that is different from the *dpy-10*, *osr-1* and *ptr-6* alleles. No cellular functions or developmental roles of C34C12.2 have been reported until now. Protein homology search by Position-Specific Iterated BLAST (National Center for Biotechnology Information, Bethesda, MA, USA) found a similarity between C34C12.2 and *Saccharomyces cerevisiae* Net1 (Figure S3). Net1 is a core subunit of the regulator of nucleolar silencing and telophase exit (RENT) complex (Straight *et al*., 1999). It tethers the RENT complex to rDNA for silencing by NAD-dependent deacetylase Sir2, another component of the complex, in the nucleolus. In order to examine whether C34C12.2 is localized in the nucleolus of germ cells, we created a transgenic strain that expressed GFP-fused C34C12.2 by MosSCI. As shown in Figure 4, C34C12.2 was predominantly localized in the nucleolus. Consistent to its potential role in germline development, C34C12.2 was detected in germ cells, oocytes and sperm, in addition to the hypodermis and the intestine. 

### **Knockdown of *nrde-2* restores fertility to *xrn-2ts*** ^*germ*^ **animals**

Our previous study failed to find C34C12.2 in XRN-2-containing complexes purified from whole-worm lysates (Miki *et al*., 2014b), raising the possibility that the two proteins function without stable physical interaction in germ cell nuclei. Wan and colleagues identified C34C12.2 in NRDE-2-containing complexes by immunoprecipitation mass spectrometry, although its function was not addressed in the study (Wan *et al*., 2020). NRDE-2 is an effector protein in the nuclear RNAi pathway. An Argonaute protein, NRDE-3 in the soma or HRDE-1 in the germline, bound by an endogenous siRNA species 22G RNA, translocates from the cytoplasm to the nucleus, where it recruits NRDE-1, -2 and -4 to the nascent pre-mRNA to inhibit elongation of RNA polymerase II and to deposit repressive histone H3K9 trimethylation marks (Guang *et al*., 2008, 2010; Burkhart *et al*., 2011; Ashe *et al*., 2012; Buckley *et al*., 2012; Luteijn *et al*., 2012). This pathway is particularly important to maintain germline integrity by silencing transposons, regulating gene expression and promoting epigenetic inheritance. Interestingly, the same machinery functions to repress expression of pre-rRNA (Zhou *et al*., 2017; Liao *et al*., 2021). If C34C12.2 functions in complex with NRDE-2 to counteract the role of XRN-2 directly or indirectly in germ cells, knockdown of *nrde-2* should restore fertility to *xrn-2ts* ^*germ*^ animals. Indeed, *xrn-2ts* ^*germ*^ animals depleted of NRDE-2 regained fertility (Figure 2B).

## Discussion

XRN-2 is a multifunctional protein that is involved in various RNA-processing pathways. Therefore, a mutagenesis screen is unlikely to isolate an allele that suppress all developmental defects caused by XRN-2 inactivation. An allele that can suppress sterility may not be sufficient for survival and maintenance of *xrn-2* mutant animals, if it fails to suppress the larval arrest phenotype, for example. We overcame this issue by restricting a target of screening to a single developmental process, namely germline development. The *xrn-2ts* ^*germ*^ conditional mutant was created by expressing a functional *xrn-2* transgene in the somatic cells and the embryo of the *xrn-2ts* mutant. A promoter of *dpy-18* was used to drive expression of *xrn-2::gfp* in somatic cells of larvae. Consistent to the previous report of *dpy-18* expression (Hill *et al*., 2000), XRN2-GFP was detected in the hypodermis of the larvae, while missing in some somatic tissues such as the intestine. Nevertheless, the mutant animals were able to develop to adult at an elevated temperature without showing somatic phenotypes such as larval arrest, a molting defect and vulval bursting, which had been reported for *xrn-2* (Frand *et al*., 2005; Miki *et al*., 2014a). Although *xrn-2* is expressed ubiquitously (Miki *et al*., 2014a), its activity in the hypodermis might be sufficient for somatic development of larvae. If so, a soma specific *xrn-2* conditional mutant could be created by expressing functional *xrn-2* in the embryo and the germline of the *xrn-2ts* mutant using appropriate promoters, which would function as a useful tool to dissect the roles of XRN-2 in somatic development of larvae. Our approach to create a spatially restricted conditional mutant would be useful to dissect a gene that has multiple essential functions in different tissues or cell-types. Although this method requires a conditional allele and has limited versatility as compared to the auxin-inducible degron system (Nishimura *et al*., 2009; Zhang *et al*., 2015; Negishi *et al*., 2022), it has advantages of low-cost and the ease of control, particularly in long-run experiments such as a genetic screen in this study.

Our results indicate that elevated accumulation of glycerol restores fertility to *xrn-2ts* ^*germ*^ animals by other means than stabilizing the mutant XRN-2 protein. A previous study suggests that increase in glycerol accumulation may function as an adaptive response to osmotic stress in the *C. elegans* germline to maintain the quality of germ cells and oocytes (Davis *et al*., 2017). This is consistent with the observation that animals that lack *gpdh-1* and *gpdh-2* genes showed reduced brood size as compared to wild-type animals under osmotic stress (Lamitina *et al*., 2006). Perhaps XRN-2 functions to maintain proper osmolality in the germline through an unknown mechanism. If so, high levels of glycerol could function as a chemical chaperon to protect XRN-2-inactivated germ cells from osmotic stress by stabilizing proteins and other structures. However, note that we cannot formally exclude the possibility that stabilization of mutant XRN-2 by glycerol is responsible for the phenotypic rescue. If somatic development of *C. elegans* larvae requires higher activity of XRN-2 than germline development, for instance, stabilization of mutant XRN-2 by glycerol might be merely insufficient for rescue of *xrn-2ts* animals from larval arrest.

*C34C12.2* is unique among the genes identified in our screen on the point that it does not affect *gpdh-1* expression. C34C12.2 is partially homologous to *S. cerevisiae* Net1, which tethers the RENT complex to rDNA for silencing (Straight *et al*., 1999). We found that depletion of NRDE-2, a putative interacting partner of C34C12.2, restored fertility to *xrn-2ts* ^*germ*^ animals. NRDE-2 functions an effector of the nuclear RNAi machinery, which negatively regulates expression of pre-mRNA and pre-rRNA (Guang *et al*., 2010; Liao *et al*., 2021). From these results and its predominant localization in the nucleolus, we speculate that C34C12.2, by interacting with the nuclear RNAi machinery through NRDE-2, represses expression of pre-rRNA and that loss of its function results in elevation of rRNA levels. Thus, the *C34C12.2* loss-of-function allele may compensate for a decrease in rRNA accumulation that is caused by XRN-2 inactivation. If so, rRNA maturation might be an essential function of XRN-2 in germline development. Further study on interaction between C34C12.2 and NRDE-2 and the impact of C34C12.2 dysregulation on rRNA accumulation will shed light on the roles of C34C12.2 and XRN-2 in germline development.

## Supplementary material

The following online material is available for this article:

Table S1 - Worm strains.

Table S2 - 
*gpdh-1* RT-qPCR data.

Figure S1 - Incubation with glycerol did not restore fertility to *xrn-2ts* ^*germ*^ animals.

Figure S2 - Knockdown of *dpy-10*, *osr-1* or *ptr-6* did not rescue *xrn-2ts* animals from larval arrest.

Figure S3 - C34C12.2 shows homology to *S. cerevisiae* Net1.
