# Supplementary material for: A germline-targeted genetic screen for xrn-2 suppressors identifies a novel gene C34C12.2 in Caenorhabditis elegans
Source: Genet Mol Biol. 2023 May 15;46(2):e20220328. doi: 10.1590/1678-4685-GMB-2022-0328 (PMC10202090; doi:10.1590/1678-4685-GMB-2022-0328)
Supplement: Table S1 - [file 1415-4757-GMB-46-02-e20220328-s1.pdf]

Genetics and Molecular Biology
https://doi.org/10.1590/1678-4685-GMB-2022-0328

Supplementary Material to "A germline-targeted genetic screen for *xrn-2* suppressors identifies a novel gene *C34C12.2* in *Caenorhabditis elegans*"

Table S1 – Worm strains.

Strain name	Genotype	Locus of single copy insertion
HW1660	xrn-2(xe31) II	
HW1682	xrn-2(xe31) II; xeSi217[Pdpy-18::xrn-2(CO)::GFP::his::flag::xrn-2 3'UTR, unc-119(+)] V	oxTi365
HW1714	xeSi219[Ppes-2.1::xrn-2(CO)::GFP::his::flag::xrn-2 3'UTR, unc-119(+)] I	ttTi4348
HW1715	xeSi219[Ppes-2.1::xrn-2(CO)::GFP::his::flag::xrn-2 3'UTR, unc-119(+)] I; xrn-2(xe31) II; xeSi217[Pdpy-18::xrn-2(CO)::GFP::his::flag::xrn-2 3'UTR, unc-119(+)] V	ttTi4348, oxTi365
TSM19	osr-1(kid1[Q239*]) I; xeSi219[Ppes-2.1::xrn-2(CO)::GFP::his::flag::xrn-2 3'UTR, unc-119(+)] I; xrn-2(xe31) II; xeSi217[Pdpy-18::xrn-2(CO)::GFP::his::flag::xrn-2 3'UTR, unc-119(+)] V	ttTi4348, oxTi365
TSM20	xeSi219[Ppes-2.1::xrn-2(CO)::GFP::his::flag::xrn-2 3'UTR, unc-119(+)] I; xrn-2(xe31) II; C34C12.2(kid2[splicing-affected]) III; xeSi217[Pdpy-18::xrn-2(CO)::GFP::his::flag::xrn-2 3'UTR, unc-119(+)] V	ttTi4348, oxTi365
TSM21	xeSi219[Ppes-2.1::xrn-2(CO)::GFP::his::flag::xrn-2 3'UTR, unc-119(+)] I; ptr-6(kid4[G223E]) II; xrn-2(xe31) II; xeSi217[Pdpy-18::xrn-2(CO)::GFP::his::flag::xrn-2 3'UTR, unc-119(+)] V	ttTi4348, oxTi365
TSM23	xeSi219[Ppes-2.1::xrn-2(CO)::GFP::his::flag::xrn-2 3'UTR, unc-119(+)] I; dpy-10(kid6[G131R])II; xrn-2(xe31) II; xeSi217[Pdpy-18::xrn-2(CO)::GFP::his::flag::xrn-2 3'UTR, unc-119(+)] V	ttTi4348, oxTi365
TSM25	kidSi3[PC34C12.2::flag::gfp::C34C12.2::C34C12.2 3'utr] II	ttTi5605
RB1032	osr-1(ok959) I	
TSM55	osr-1(ok959) I; xrn-2(xe31) II	
