# Supplementary material for: A germline-targeted genetic screen for xrn-2 suppressors identifies a novel gene C34C12.2 in Caenorhabditis elegans
Source: Genet Mol Biol. 2023 May 15;46(2):e20220328. doi: 10.1590/1678-4685-GMB-2022-0328 (PMC10202090; doi:10.1590/1678-4685-GMB-2022-0328)
Supplement: Table S2 - [file 1415-4757-GMB-46-02-e20220328-s2.pdf]

Genetics and Molecular Biology
https://doi.org/10.1590/1678-4685-GMB-2022-0328

Supplementary Material to "A germline-targeted genetic screen for *xrn-2* suppressors identifies a novel gene *C34C12.2* in *Caenorhabditis elegans*"

Table S2 – *gpdh-1* RT-qPCR data.

Treatment	Repeat 1	Repeat 2	Repeat 3	Repeat 4	Repeat 5	Mean	SEM	p-value
Mock	1	1	1	1	1	1	0	0
dpy-10(RNAi)	14.84098	26.34617	8.223292	17.85773	19.46333	17.3463	2.960126	0.000279
osr-1(RNAi)	3.89394	5.619918	2.381454	5.862968	14.66214	6.484085	2.139552	0.01674
ptr-6(RNAi)	4.983817	15.98594	3.04262	10.12917	17.19792	10.26789	2.836077	0.005696
C34C12.2(RNAi)	0.691002	0.793935	1.036331	1.888086	1.47115	1.176101	0.223034	0.226272
