# Supplementary material for: A germline-targeted genetic screen for xrn-2 suppressors identifies a novel gene C34C12.2 in Caenorhabditis elegans
Source: Genet Mol Biol. 2023 May 15;46(2):e20220328. doi: 10.1590/1678-4685-GMB-2022-0328 (PMC10202090; doi:10.1590/1678-4685-GMB-2022-0328)
Supplement: Figure S1 - [file 1415-4757-GMB-46-02-e20220328-s10.pdf]

Genetics and Molecular Biology
https://doi.org/10.1590/1678-4685-GMB-2022-0328

Supplementary Material to "A germline-targeted genetic screen for *xrn-2* suppressors identifies a novel gene *C34C12.2* in *Caenorhabditis elegans*"

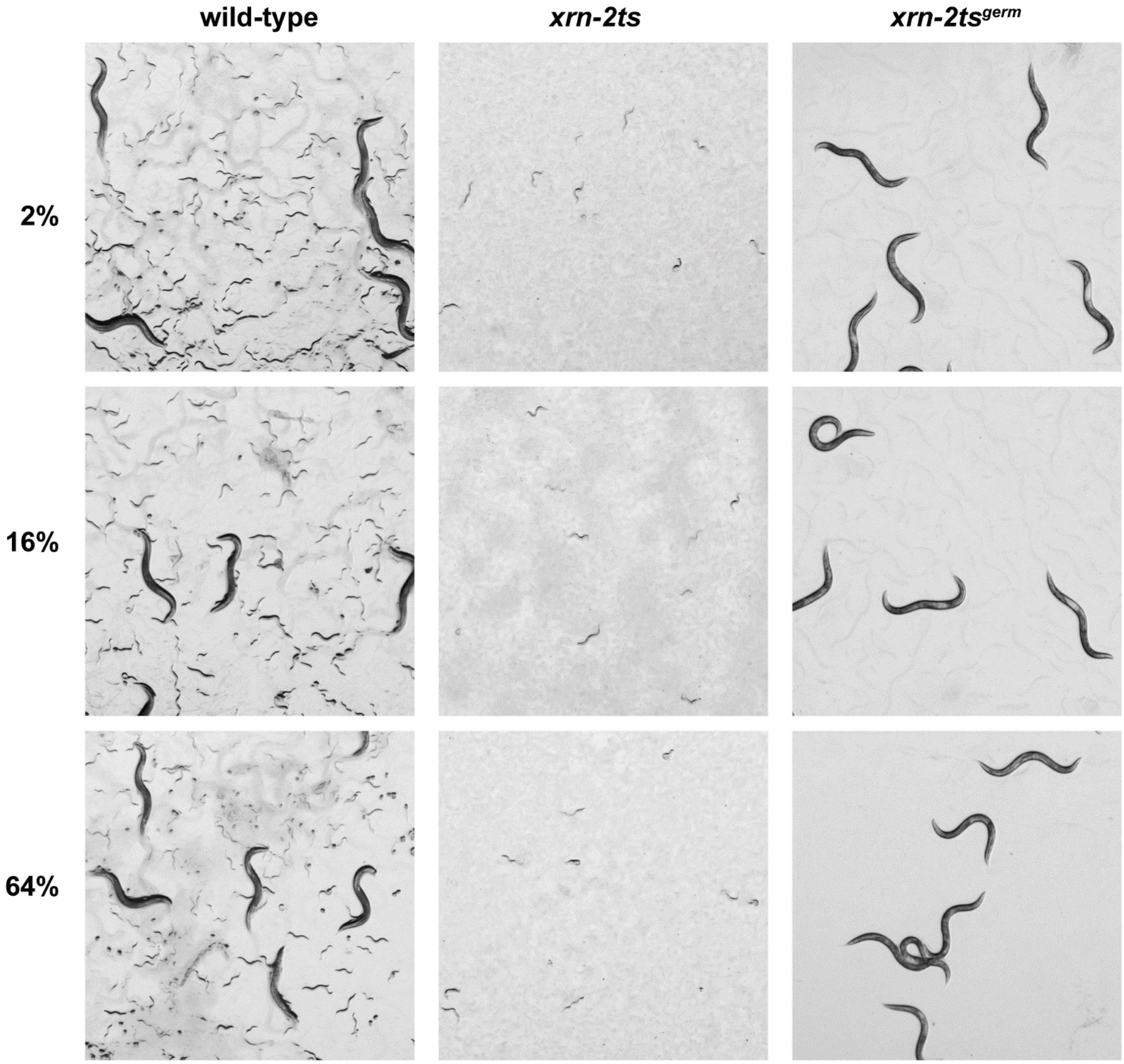

Figure S1 - Incubation with glycerol did not restore fertility to *xrn-2ts^germ* animals. Wild-type, *xrn-2ts* and *xrn-2ts^germ* animals were incubated on plates with 2%, 16% or 64% glycerol at 26°C from L1 stage for 72 hours and observed by stereomicroscopy at the same magnification. *xrn-2ts^germ* animals developed to adult but were sterile, while wild-type animals reproduced. *xrn-2ts* animals ceased development at the L1 stage.
