# Supplementary material for: A germline-targeted genetic screen for xrn-2 suppressors identifies a novel gene C34C12.2 in Caenorhabditis elegans
Source: Genet Mol Biol. 2023 May 15;46(2):e20220328. doi: 10.1590/1678-4685-GMB-2022-0328 (PMC10202090; doi:10.1590/1678-4685-GMB-2022-0328)
Supplement: Figure S2 - [file 1415-4757-GMB-46-02-e20220328-s20.pdf]

Genetics and Molecular Biology
https://doi.org/10.1590/1678-4685-GMB-2022-0328

Supplementary Material to "A germline-targeted genetic screen for *xrn-2* suppressors identifies a novel gene *C34C12.2* in *Caenorhabditis elegans*"

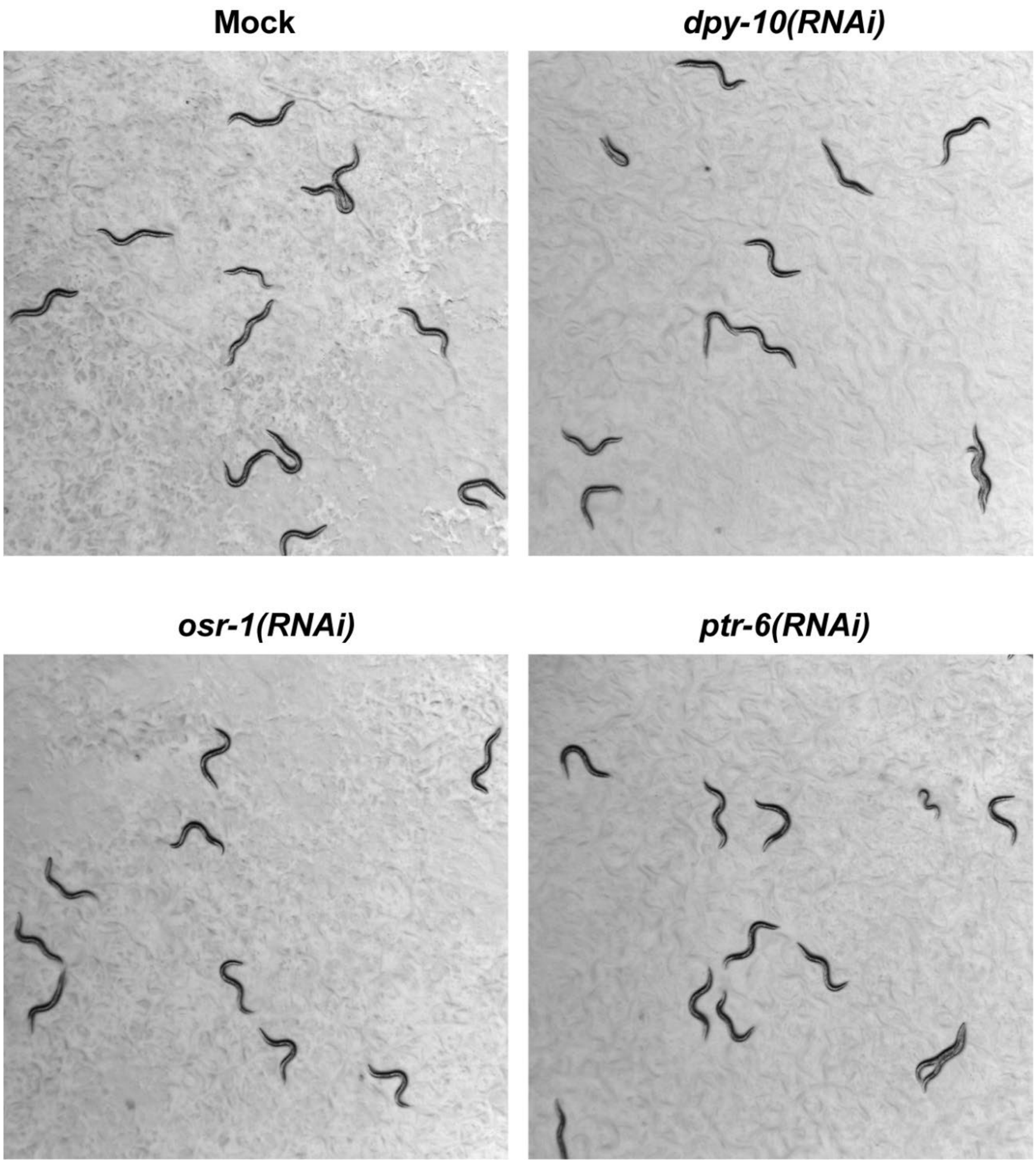

Figure S2 - Knockdown of *dpy-10*, *osr-1* or *ptr-6* did not rescue *xrn-2ts* animals from larval arrest. *xrn-2ts* animals were exposed to mock RNAi or RNAi for indicated genes from the L1 stage for 48 hours at 25°C and observed by stereomicroscopy at the same magnification. All animals ceased development as larvae.
