# Supplementary material for: A germline-targeted genetic screen for xrn-2 suppressors identifies a novel gene C34C12.2 in Caenorhabditis elegans
Source: Genet Mol Biol. 2023 May 15;46(2):e20220328. doi: 10.1590/1678-4685-GMB-2022-0328 (PMC10202090; doi:10.1590/1678-4685-GMB-2022-0328)
Supplement: Figure S3 - [file 1415-4757-GMB-46-02-e20220328-s30.pdf]

Genetics and Molecular Biology

https://doi.org/10.1590/1678-4685-GMB-2022-0328

Supplementary Material to "A germline-targeted genetic screen for *xrn-2* suppressors identifies a novel gene *C34C12.2* in *Caenorhabditis elegans*"

Net1p [Saccharomyces cerevisiae YJM1399]

Sequence ID: AJV44886.1 **Length:** 1195 **Number of Matches:** 1

Range 1: 775 to 1189 GenPept Graphics ▼ Next Match ▲ Previous Match

Score	Expect	Identities	Positives	Gaps
55.1 bits(131)	1e-06	99/446(22%)	165/446(36%)	49/446(10%)

```
Query  92     ARQAKISQNLMKTSETASTSSPVRQQVKTEIVPRVS--QRTIVKTQALPRVNA---PVLS  146
              +++  +     KTS+++ T S + +Q+     P ++  Q    K + P   A   PV+S
Sbjct  775    SQKKSVVSESFKTSQSSVTDSKISEQMAKSFYPNLNKKQNEATKVETKPATQASFFPVVS  834

Query  147    SVPAKNPLPPKFVYINTAGLKRKRDDDKNTPSTSNSITLNPSSRGDQTQFIVHLQRTIER  206
                P+          + T G     ++        N   +   ++ +  Q     + T  R
Sbjct  835    GAPS----------VATKGTSSFNEE-------GNRKNVKTKAKNESAQIDRQQKETTSR  877

Query  207    LEKEKAALT--EKLTMREDEIKVFSVEFVNIQKENVK--LMKENKAKESQISNQSIQVRN  262
              +   K+A    E L  + +  K       NIQ  N K  L ++  +K  Q+S + +++ +
Sbjct  878    VTDLKSANIGGEDLNKKAEGSKEPEKASANIQDANDKNNLKEKEDSKSKQVSQKKLKMTD  937

Query  263    SWKFAD--LFKKELQKSRKDVSEVKWKIDKIEKKVGI----KKPTPRKKPDVKLI--NPE  314
                K  +  L K       KD+ + K+   K     GI    K  +     D      + E
Sbjct  938    HLKEGNVQLPKPSANDKLKDL-KAKFTNSKTLVPPGIISNEKNNSSANDDSSSSGSSTE  996

Query  315    DISLMSDRTQDGEDSSDFGSPLAKYLKPDQPSTSSACYGKPFYFESTSSSSRKPITASPG  374
              D S  S  + D E S+   +       P +P  SS+        E+ S S  K I A+P
Sbjct  997    DESSSSSSSSDEETSTSRKARRVVVNTPREPVRSSS------KIEAPSPSVNKKINATPD  1050

Query  375    PPGRTQISDQLNTGEVRYVVNSGKPFNFSSESNSRNLKLIPGYIKRPEFRYIKPEGFTSA  434
                  TQ+ D  +   V+    S    N SS  +    K+ P      +   +   G
Sbjct  1051   KIPVTQLMDMSSPPSVKSKTTS----NPSSILHDLPRKVRPSLSSLSD---LVSRGIPDV  1103

Query  435    SYKAQSEGMSSFLKTGSSATPENSKKSAHFDMPD-ISSTPYKSHVVVESDEMNSSSSTIG  493
                K       S  K  SS+  E+S  S      D +S +   S    +SD+   SS
Sbjct  1104   KEKTSKSNEKSQTKASSSSDDESSSDSDSNSSSDSVSDSSSDSKSESDSDDSGDSSDDGK  1163

Query  494    GFESEKKDNGALGSQKSPMPDIATAL  519
               F S K  + ALG +K P    A+ +
Sbjct  1164   SFISAKSASAALGKKKKPSGGFASLI  1189
```

Figure S3 - C34C12.2 shows homology to S. cerevisiae Net1. The full-length sequence of the C34C12.2 protein (NCBI accession ID: NP_497712) was analyzed by PSI-BLAST with default algorithm parameters except "no adjustment" selected for compositional adjustments.
